# Hypsugopoxvirus: A Novel Poxvirus Isolated from *Hypsugo savii* in Italy

**DOI:** 10.3390/v11060568

**Published:** 2019-06-19

**Authors:** Davide Lelli, Antonio Lavazza, Alice Prosperi, Enrica Sozzi, Francesca Faccin, Laura Baioni, Tiziana Trogu, Gian Luca Cavallari, Matteo Mauri, Anna Maria Gibellini, Chiara Chiapponi, Ana Moreno

**Affiliations:** 1 Istituto Zooprofilattico Sperimentale della Lombardia e dell’Emilia Romagna, Via Bianchi 9, 25124 Brescia, Italy; antonio.lavazza@izsler.it (A.L.); alice.prosperi@izsler.it (A.P.); enrica.sozzi@izsler.it (E.S.); francesca.faccin@izsler.it (F.F.); laura.baioni@izsler.it (L.B.); tiziana.trogu@izsler.it (T.T.); chiara.chiapponi@izsler.it (C.C.); anamaria.morenomartin@izsler.it (A.M.); 2 Wildlife Rehabilitation Center WWF of Valpredina via Pioda n.1, 24060 Cenate Sopra (BG), Italy; clivetorzi@gmail.com (G.L.C.); info@oasivalpredina.it (M.M.); pipistrelli@valpredina.eu (A.M.G.)

**Keywords:** bats, poxvirus, Italy

## Abstract

Interest in bat-related viruses has increased considerably during the last decade, leading to the discovery of a rising number of new viruses in several bat species. *Poxviridae* are a large, diverse family of DNA viruses that can infect a wide range of vertebrates and invertebrates. To date, only a few documented detections of poxviruses have been described in bat populations on three different continents (America, Africa, and Australia). These viruses are phylogenetically dissimilar and have diverse clinical impacts on their hosts. Herein, we report the isolation, nearly complete genome sequencing, and annotation of a novel poxvirus detected from an insectivorous bat (*Hypsugo savii*) in Northern Italy. The virus is tentatively named Hypsugopoxvirus (HYPV) after the bat species from which it was isolated. The nearly complete genome size is 166,600 nt and it encodes 161 genes. Genome analyses suggest that HYPV belongs to the Chordopoxvirinae subfamily, with the highest nucleotide identity (85%) to Eptesipoxvirus (EPTV) detected from a microbat *Eptesicus fuscus* in WA, USA, in 2011. To date, HYPV represents the first poxvirus detected in bats in Europe; thus, its viral ecology and disease associations should be investigated further.

## 1. Introduction

Poxviruses are dsDNA viruses with large genomes (130 to 360 kb) that belong to the family Poxviridae. The family is divided into the Entomopoxvirinae and the Chordopoxvirinae subfamilies of viruses, which infect insects and vertebrates, respectively. According to the International Committee on Taxonomy of Viruses (ICTV) 2017 Release [1], 11 genera have been created to classify Chordopoxviruses (*Avipoxvirus, Capripoxvirus, Centapoxvirus, Cervidpoxvirus, Crocodylidpoxvirus, Leporipoxvirus, Molluscipoxvirus, Orthopoxvirus, Parapoxvirus, Suipoxvirus,* and *Yatapoxvirus*), but other viruses remain unclassified and new genera are likely to be recognized in the future. Poxviruses show a diverse host range, with some viruses having wide host tropism (e.g., Orthopoxviruses) and thus being consequently associated with greater zoonotic risks [2], and others having strict host specificity.

In recent decades, bats have been increasingly recognized as reservoirs of emerging viral infections, which has important ramifications for animal and public health [3]. However, the majority of bat-borne viruses that can cause severe diseases in humans and other mammals, do not cause apparent clinical signs in bats. Consequently, it has been assumed that bats may have a “special” relationship with viruses based on physiological, ecological, evolutionary, and/or immunological aspects, which allow them to act as special viral reservoirs with exaggerated viral richness [4,5,6,7].

Currently, four poxviruses from the Microchiroptera and Macrochiroptera suborders have been detected in bat populations on three continents (America, Africa, and Australia) [8]. Specifically, Eptesipoxvirus (EPTV) was isolated in North America in 2011 from *Eptesicus fuscus* [9,10]; Eidolon helvum poxvirus 1 (EHPV1) was detected in West Africa in 2009 from *Eidolon helvum* [11]; the Pteropox virus (PTPV) was identified in Northwestern Australia in 2015 from *Pteropus scapulatus* [12]; and a fourth poxvirus was also identified in South Australia from *Miniopterus schreibersii bassanii* in 2009 [13]. It is remarkable that these viruses are phylogenetically divergent and are associated with variable clinical manifestations.

Virological investigations focused on poxviruses in bat populations may have a positive impact for future ecological studies of bat–pathogen interactions. Moreover, from the perspective of the One Health approach, bats could benefit from these studies, since European bat populations are currently undergoing a global decline that could be linked with so far overlooked viral infections.

In this study, we report the isolation, nearly complete genomic sequencing, and annotation of a novel poxvirus detected from an insectivorous bat *(Hypsugo savii)* in Northern Italy. The virus was tentatively named Hypsugopoxvirus (HYPV), according to the bat species from which it was isolated. Phylogenetic analyses suggest that HYPV belongs to the Chordopoxvirinae subfamily, revealing the highest similarity (85%) with Eptesipoxvirus (EPTV) detected from the microbat *Eptesicus fuscus* in WA, USA in 2011, which is associated with bat necrosuppurative osteomyelitis in multiple joints. HYPV is the first poxvirus detected in bats in Europe and its viral ecology and disease associations should be investigated further.

## 2. Materials and Methods

### 2.1. Sampling

Dead bats from different species were collected for virological investigations from wild animal rescue/rehabilitation centers in the context of a general surveillance project that has been implemented in Northern Italy since 2009–2010, which focuses on the detection of emerging bat viruses [14,15,16]. The bats were taxonomically identified based on their morphologic characteristics, according to the European bat identification keys [15]. The carcasses were necropsied, and tissue samples were collected for further laboratory exams, particularly for viral detection and isolation.

### 2.2. Virological Analysis

After necropsy, organ samples (lungs, heart, kidney, brain, and intestines) were mechanically homogenized in minimal essential medium (1 g/10 mL), which contained antibiotics. They were then centrifuged at 3000 g for 15 min. Samples were inoculated in confluent monolayers of VERO and MARC 145 cells (African green monkey), incubated at 37 °C with 5% CO_2_ and observed daily for seven days to assess their cytopathic effects (CPEs). In the absence of CPEs, the cryolysates were sub-cultured twice onto fresh monolayers. Cell culture supernatants showing CPE were partially purified by ultracentrifugation at 35,000 rpm for 2 h (rotor TST41 Kontron) through a 25% (*w*/*w*) sucrose cushion, and the pellet was re-suspended in PBS. This antigen was kept at −70 °C and then submitted for viral identification with the NGS approach and negative-staining electron microscopy (nsEM) by using the Airfuge (Beckman Instruments, Palo Alto, CA, USA) method [17].

### 2.3. Molecular Analysis

Viral DNA was extracted from 200 μL of positive cell culture supernatants using a BioSprint 96 One-For-All Vet Kit (Qiagen S.p.A., Milan, Italy). Sequencing libraries were made with a Nextera Flex kit (Illumina Inc. San Diego, CA, USA) in accordance with the manufacturer’s instructions. Libraries were sequenced on a MiSeq Instrument (Illumina Inc. San Diego, CA, USA) by using a MiSeq Reagent Kit v2 in a 250 cycle paired-end run. Data were assembled de novo by the CLC Genomic workbench v.11 (Qiagen S.p.A., Milan, Italy).

Genome annotation and analysis was performed with tools from the bioinformatics suite developed at the Viral Bioinformatics Resource Centre [18]. The Genome Annotation Transfer Utility (GATU) [19] uses a reference genome to automatically annotate poxvirus genes with clear orthologs in the reference. Other possible genes were presented to the annotator for further characterization and to make final annotation decisions.

## 3. Results

### 3.1. Clinical Case

The case specifically concerned a juvenile *Hypsugo savii* male that spontaneously died in a wildlife recovery center in Valpredina, Cenate Sopra (BG), Northern Italy after several weeks of hospitalization. The sick bat was originally found alive on July 17, 2017 in Telgate (Bergamo Province, Northern Italy) by a private citizen who brought it to the center. Clinically, the bat had a humerus fracture, sensory depression and a lack of appetite but normal body mass. The death occurred 54 days after admission to the center on September 9, 2017; then, the carcass was sent to the lab for necroscopy and further analyses. Pathological lesions in the internal organs indicative of infectious diseases were not observed, but a soft bone callus due to pathological healing of the humerus fracture associated with osteomalacia and calcium deficiency was detected.

### 3.2. Virus Isolation and Identification

**Figure 1:**
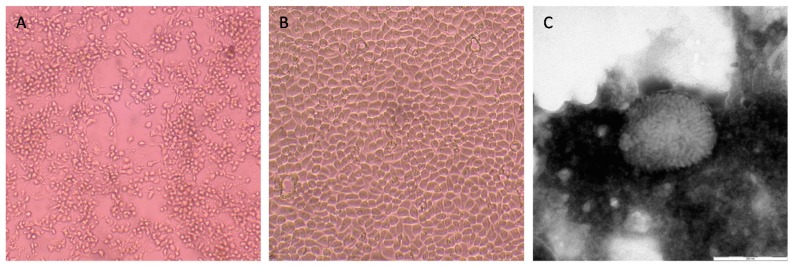
(**A**) Cytopathic effects (CPEs) of rounded cells floating in the culture medium of MARC 145 cells infected with the pool of bat organs (heart and lungs) at three days after inoculation (original magnification × 100); (**B**) mock cells (original magnification ×100); (**C**) negative-staining electron microscopy showing the presence of a virion morphologically related to the *Orthopoxvirus* genus from the MARC 145 cell culture.

A virus was isolated on MARC 145 cells inoculated with the organ pool composed of the bat’s heart and lungs. The CPE occurred on the third day post-inoculation during the second passage and was characterized by a diffused degeneration of a monolayer with rounded cells floating in the culture medium (Figure 1A,B). The cell culture supernatant showing CPE was submitted to the NGS in order to identify and characterize the unknown isolate. Furthermore, nsEM performed on the purified and concentrated antigen revealed the presence of viral particles that unequivocally morphologically resembled those belonging to the genus *Orthopoxvirus* (Figure 1C). The virus was tentatively named Hypsugopoxvirus (HYPV), according to the bat species from which it was isolated. Table 1 summarizes the basic information on the HYPV identified in this study in comparison with all known poxviruses detected to date in bats worldwide.

### 3.3. Genome Characterization

After NGS sequencing, the nearly complete viral genome of a poxvirus was obtained from one contig of 166,600 nucleotides originating from 85,678 reads with an average coverage of 118.53. The nearly full genome sequence of the viral strain was determined and compared with those of other members of the Poxviridae family available on GenBank. For the nearly complete viral genome sequencing, BLAST analysis revealed the highest nucleotide identity (85%) to the Eptesipoxvirus (EPTV) strain “Washington”, a member of the Chordopoxvirinae subfamily identified in microbats in the USA (Table 2). The nearly complete genome sequence for HYPV was submitted to GenBank under accession number MK860688.

A conservative approach was taken for genome annotation to avoid over-annotating open reading frames (ORFs) that were unlikely to represent functional genes. ORFs less than 50 codons or overlapping by more than 25% with well-characterized genes were not considered for annotation unless supported by other evidence. A total of 161 genes were annotated for HYPV, showing a percentage value of nt identity with its closest related virus EPTV ranging from 42.5% for the HYPV-2 gene (serpin 2) to 100% for the HYPV-90 gene (VLTF-3) (Table 3).

When the seven conserved genes—*RPO147*, *RAP94*, mRNA capping enzyme large subunit, P4a precursor, *RPO132*, *VETF-L*, and DNA primase—were considered individually, the value of nt similarity with EPTV ranged from 90.5% to 98.5%. The above conserved genes that have been used for phylogenetic analysis in previous studies [10,12] are presented in bold in Table 3.

HYPV showed nucleotide divergence from its closest relative, EPTV. The smaller genome size with 166,600 nt encoding 161 genes for HYPV in comparison to 176,688 nt and 191 genes for EPTV, is likely due to the omission of the ITRs from the analysis and therefore, is not possible to establish the exact length of its the viral genome. Two ORFs (HYPV-24 and HYPV-25, Table 3), whose function is still unknown, appear to be unique to HYPV.

## 4. Discussion

The potential zoonotic risks associated with bats and their fascinating and special relationship with viruses have attracted the attention of many researchers worldwide. Consequently, general and target surveillance on bat populations has increased in the last decade with the purpose of clarifying the genetic diversity of bat-associated viruses as well as acquiring comprehensive information on bat–pathogen interactions. In fact, viral disease prevention and biological conservation issues could both benefit from such research.

Virological surveillance of bat populations in Italy is a relative novelty and has only recently been extensively applied, but almost immediately, a great heterogeneity of virus identifications has been observed. Viruses belonging to several viral families, such as Reoviridae [14], Coronaviridae [15,20,21,22,23,24], Paramyxoviridae [24], Rhabdoviridae [16,25], and Astroviridae [26], have been detected, allowing the identification of some novel/previously unknown viral agents. The results of the general surveillance of bats, which have been randomly applied so far as pilot virus discovery studies, may drive future activity to more specific longitudinal and target studies aimed at understanding the epidemiology of potential new pathogens.

In this study, a novel poxvirus, HYPV, was detected from the microbat *Hypsugo savii* in Italy. This likely represents the first poxvirus detection in bats in Europe. In fact, only four poxviruses have been documented to date in bat populations worldwide, and these and these have diverse and somehow incomplete descriptions, with just some common aspects. Firstly, EHPV1 was detected in 2009 with a high-prevalence in throat swabs from apparently healthy African megabats (*Eidolon helvum*), and metagenomic analysis identified poxvirus sequences that were most closely related with Molluscum contagiosum (MOCV), a human-only pathogen [11]. In the same year of 2009, another bat poxvirus was incidentally detected in South Australia during the investigation of an outbreak of parasitic skin disease in a population of the microbat species, *Miniopterus schreibersii bassanii*. In one of the twenty-one bats examined, an independent (non-nematode-associated) lesion containing intracytoplasmic inclusion bodies indicative of poxvirus infection was observed, and this was confirmed with electron microscopy [13]. Between 2009 and 2011, EPTV was detected in adult big brown bats (*Eptesicus fuscus*) with severe joint disease (tenosynovitis and osteoarthritis) at a wildlife center in Northwestern United States. Phylogenetic analysis revealed that Eptesipoxvirus is most closely related to the Cotia virus, a virus detected in sentinel suckling mice in Sao Paulo, Brazil in 1961 [27,28]. PTPV was detected from an Australian little red flying fox (*Pteropus scapulatus*) that died following entrapment on a fence. Post-mortem examination revealed multiple nodules on the wing membranes. Phylogenetic analysis indicated that PTPV is not closely related to any other poxvirus isolated from bats or other species, and that it likely should be placed in a new genus [12].

It is noteworthy that PTPV and EHPV were isolated from megabat hosts (*Pteropus scapulatus* and *Eidolon helvum*, respectively), whereas EPTV and HYPV were isolated from microbats (*Eptesicus fuscus* and *Hypsugo savii*, respectively). While EHPV was detected in apparently healthy bats, the other viruses were identified in sick bats and their association with the pathological condition was assumed. Specifically, clinical symptoms of EPTV in *Eptesicus fuscus* manifested in the form of joint swelling and increased lethargy [10]. PTPV-infected *Pteropus scapulatus* presented vesicular to nodular skin lesions on the wing membranes that are typical of poxvirus infections [13]. HYPV was detected in a bat showing pathological healing of the humerus fracture associated with osteomalacia and calcium deficiency. Neither symptom was directly linked to fatality and thus the capability of these viruses still needs to be ascertained, including the role of HYPV in causing deadly disease in bats.

The results of our study indicate that HYPV presents the typical morphology of the *Orthopoxvirus* genus and that it could be isolated in cell culture. Indeed, its final identification was obtained by genomic characterization. The nearly complete genomic sequencing clearly demonstrated that HYPV is a new virus that is distantly related to its closest known relative EPTV (WA, USA, 2011) with a nucleotide identity of 85% (almost whole genome). Indeed, the percentage value of the nt identity of HYPV with EPTV ranged from 42.5% for the HYPV-2 gene (serpin 2) to 100% for the HYPV-90 gene (VLTF-3). Regarding ORFs annotation the HYPV was shown to be defective in particular in the ITR genes i.e., 12 out of 13 described in EPTV, but this should be not a real structural defect but more likely due to the omission of the ITRs from the analysis. On the contrary, two ORFs, whose function is still unknown, appear to be unique to HYPV.

To conclude, a new poxvirus, HYPV, was detected in bats in Europe and its viral ecology and disease associations should be investigated further.

## Figures and Tables

**Table 1:** Basic data on Hypsugopoxvirus (HYPV) in comparison with all known poxviruses detected to date in bats worldwide.

Poxvirus Strain	Host	Sample Source	Origin	Collection Date	Clinical/Post-Mortem Findings	Laboratory Outcomes	Ref.
Hypsugopox virus (HYPV) Id lab: IZSLER 251170-23/2017	*Hypsugo savii*	Pool of viscera (heart and lungs)	Europe (Italy)	2017	Humerus fracture and osteomalacia, calcium deficiency	CC, EM, nFGS (166,600 nt), GA (161 genes)	This study
Pteropox virus (PTPV)	*Pteropus scapulatus*	Wing membrane	North Western Australia (Kimberley region)	2015	Multiple nodules on the wing membranes	PGS (133,492 nt), GA (143 genes)	[12]
Eptesipox virus (EPTV) strain “Washington“	*Eptesicus fuscus*	Elbow joint	America (WA, USA)	2011	Necro-suppurative osteomyelitis in multiple joints	CC, EM, FGS (176,688 nt), GA (191 genes)	[9,10]
Eidolon helvum poxvirus 1 (EHPV1)	*Eidolon helvum*	Throat swabs	Africa (Ghana)	2009	Apparently healthy bats	PGS	[11]
NA	*Miniopterus schreibersii bassanii*	Skin biopsies	South Australia (Naracoorte)	2009	Nodular cutaneous lesions	EM	[13]

NA: not available; CC: cell culture isolation; EM: electron microscopy identification; FGS: full-genome sequence; nFGS: nearly full-genome sequence; PGS: partial genome sequence; GA: genome annotation.

**Table 2:** Highest nucleotide sequence identities for the nearly complete genome of HYPV.

% Similarity	Query Cover %	Poxvirus Strain	GenBank Accession No.	Host	Ref.
85	75	Eptesipoxvirus strain “Washington”	KY747497	*Eptesicus fuscus*	[3,4]

**Table 3:** HYPV genome annotation and nucleotide identities for each gene to the most similar strain Eptesipoxvirus (EPTV). The seven conserved genes used for phylogenetic analysis in previous studies [10,12] are presented in bold.

Gene Name	Putative Product Identity	Start	Stop	+/−	Size	% Id. to EPTV	Orthologs
HYPV-1	Hypothetical protein	87	557	−	471	58	EPTV-001
HYPV-2	Serpin 2	1037	1552	−	516	42.5	EPTV-002
HYPV-3	Hypothetical protein	1581	2261	−	681	82.4	EPTV-003
HYPV-4	IL-1 receptor-like protein	2309	3316	−	1008	65.1	EPTV-004
HYPV-5	Hypothetical protein	3356	3835	−	480	88.8	EPTV-005
HYPV-6	Tyrosine protein kinase-like protein	3872	4774	−	903	91.7	EPTV-006
HYPV-7	ER-localized apoptosis regulator	4842	5522	−	681	63.6	EPTV-007
HYPV-8	Hypothetical protein	7002	7481	−	480	80.5	EPTV-008
HYPV-9	Ankyrin repeat-containing protein, host range	8141	9826	−	1686	63.0	EPTV-010
HYPV-10	Monoglyceride lipase	11,053	11,913	−	861	93.1	EPTV-014
HYPV-11	Secreted EGF-like growth factor	12,340	12,588	−	249	62.4	EPTV-015
HYPV-12	Anti-apoptotic factor	12,594	13,100	−	507	65.7	EPTV-016
HYPV-13	dUTPase	13,144	13,569	−	426	87.2	EPTV-017
HYPV-14	IFN-inducible protein	13,597	14,004	−	408	83.7	EPTV-018
HYPV-15	Ribonucleotide reductase small subunit	14,060	15,034	−	975	93.8	EPTV-019
HYPV-16	F5L membrane protein	15,075	16,139	−	1065	68.4	EPTV-020
HYPV-17	Cytoplasmic protein	16,687	16,869	−	183	71.4	EPTV-023
HYPV-18	S–S bond formation pathway protein	17,361	18,008	−	648	92.6	EPTV-025
HYPV-19	Ser|Thr protein kinase	17,998	19,314	−	1317	94.7	EPTV-026
HYPV-20	RhoA signaling inhibitor, virus release protein	19,334	20,626	−	1293	88.0	EPTV-07
HYPV-21	EEV maturation protein	20,659	22,602	−	1944	89.0	EPTV-028
HYPV-22	Palmitylated EEV membrane glycoprotein	22,640	23,755	−	1116	98.9	EPTV-029
HYPV-23	Hypothetical protein	23,781	24,008	−	228	67.1	EPTV-031
HYPV-24	Hypothetical protein	24,050	24,250	−	201	97.0	Unique to HYPV
HYPV-25	Hypothetical protein	24,471	24,917	−	447	92.6	Unique to HYPV
HYPV-26	Conserved non-functional serine recombinase	24,992	25,654	−	663	78.8	EPTV-033
HYPV-27	DNA-binding phosphoprotein	25,714	26,052	+	339	86.7	EPTV-034
HYPV-28	Poly (A) polymerase catalytic subunit	26,046	27,461	−	1416	92.6	EPTV-035
HYPV-29	IEV morphogenesis	27,478	29,676	−	2199	93.3	EPTV-036
HYPV-30	RNA polymerase subunit	29,733	30,455	−	723	93.8	EPTV-038
HYPV-31	IMV protein, virion morphogenesis	30,760	32,463	+	1704	95.8	EPTV-039
HYPV-32	ER-localized membrane protein, virion core protein	32,490	33,302	+	813	95.6	EPTV-040
HYPV-33	DNA polymerase	33,299	36,319	−	3021	93.8	EPTV-041
HYPV-34	Sulfhydryl oxidase (FAD-linked)	36,352	36,642	+	291	96.9	EPTV-042
HYPV-35	Virion core protein	36,645	37,055	−	411	87.9	EPTV-043
HYPV-36	Virulence, modulates Raf|MEK|ERK pathway	37,039	39,117	−	2079	91.9	EPTV-044
HYPV-37	Nonessential glutaredoxin	39,173	39,487	−	315	91.3	EPTV-045
HYPV-38	DNA-binding core protein	39,613	40,545	−	933	90.3	EPTV-046
HYPV-39	IMV membrane protein	40,546	40,767	−	222	83.6	EPTV-047
HYPV-40	ssDNA-binding phosphoprotein	40,768	41,577	−	810	87.5	EPTV-048
HYPV-41	Ribonucleotide reductase large subunit	41,640	43,925	−	2286	95.2	EPTV-049
HYPV-42	IMV protein (VP13)	43,966	44,202	−	237	88.5	EPTV-050
HYPV-43	Telomere-binding protein	44,220	45,371	−	1152	90.9	EPTV-051
HYPV-44	Viral core cysteine proteinase	45,364	46,650	−	1287	94.6	EPTV-052
HYPV-45	RNA-helicase, DExH-NPH-II	46,656	48,686	+	2031	94.3	EPTV-053
HYPV-46	Insulin metalloproteinase-like protein	48,678	50,465	−	1788	92.3	EPTV-054
HYPV-47	Entry|fusion complex component	50,462	50,794	−	333	97.3	EPTV-055
HYPV-48	Late transcription elongation factor (VLTF)	50,788	51,456	+	669	90.5	EPTV-056
HYPV-49	Thioredoxin-like protein	51,423	51,800	−	378	89.6	EPTV-057
HYPV-50	FEN1-like nuclease	51,803	53,140	+	1338	87.0	EPTV-058
HYPV-51	RNA polymerase subunit	53,142	53,333	+	192	96.8	EPTV-059
HYPV-52	NLPc|P60 superfamily protein	53,337	53,870	+	534	87.7	EPTV-060
HYPV-53	Virion structural phosphoprotein, early morphogenesis	53,836	54,933	−	1098	91.3	EPTV-061
HYPV-54	Late transcription factor	54,962	55,744	+	783	98.5	EPTV-062
HYPV-55	Myristylated entry|fusion protein	55,760	56,782	+	1023	93.8	EPTV-063
HYPV-56	Myristylated IMV envelope protein	56,783	57,532	+	750	96.4	EPTV-064
HYPV-57	Crescent membrane|immature virion protein	57,558	57,833	+	276	84.6	EPTV-065
HYPV-58	Internal virion protein	57,825	58,790	−	966	92.1	EPTV-066
HYPV-59	DNA-binding virion protein	58,815	59,573	+	759	98.4	EPTV-067
HYPV-60	IMV protein, entry|fusion	59,588	59,992	+	405	94.0	EPTV-068
HYPV-61	IMV membrane protein, virion morphogenesis	59,934	60,380	+	447	95.9	EPTV-069
HYPV-62	Thymidine kinase	60,402	60,932	+	531	93.8	EPTV-070
HYPV-63	Type I IFN inhibitor	61,026	61,625	+	600	73.6	EPTV-071
HYPV-64	Poly (A) polymerase small subunit	61,692	62,693	+	1002	94.3	EPTV-072
HYPV-65	RNA polymerase subunit (RPO22)	62,608	63,165	+	558	96.8	EPTV-073
HYPV-66	IMV membrane protein, entry|fusion	63,170	63,580	−	411	94.1	EPTV-074
**HYPV-67**	**RNA polymerase subunit (RPO147)**	**63,688**	**67,545**	**+**	**3858**	**98.5**	EPTV-075
HYPV-68	Tyr|Ser kinase, virus assembly, IFN-gamma inhibitor	67,542	68,060	−	519	97.7	EPTV-076
HYPV-69	Entry|fusion IMV protein	68,074	68,646	+	573	98.9	EPTV-077
HYPV-70	IMV heparin-binding surface protein	68,654	69,667	−	1014	90.6	EPTV-078
**HYPV-71**	**RNA polymerase-associated protein (RAP94)**	**69,671**	**72,058**	**−**	**2388**	**97.5**	EPTV-079
HYPV-72	Late transcription factor	72,228	72,872	+	645	71.9	EPTV-080
HYPV-73	DNA topoisomerase type I	72,894	73,829	+	936	93.9	EPTV-081
HYPV-74	Crescent membrane|immature virion protein	73,868	74,314	+	447	88.6	EPTV-082
**HYPV-75**	**mRNA capping enzyme large subunit**	**74,355**	**76,889**	**+**	**2535**	**95.7**	EPTV-083
HYPV-76	Virion core protein	76,851	77,288	−	438	89.8	EPTV-084
HYPV-77	Virion core protein	77,287	78,030	+	744	83.0	EPTV-085
HYPV-78	Uracil DNA glycosylase, DNA pol processivity factor	78,027	78,683	+	657	96.8	EPTV-086
**HYPV-79**	**NTPase, DNA primase**	**78,717**	**81,080**	**+**	**2364**	**96.6**	EPTV-087
HYPV-80	Early transcription factor small subunit (VETF-s)	81,077	82,984	+	1908	99.1	EPTV-088
HYPV-81	RNA polymerase subunit	83,017	83,532	+	516	90.1	EPTV-089
HYPV-82	Carbonic anhydrase, GAG-binding MV membrane protein	83,464	84,339	−	876	82.5	EPTV-090
HYPV-83	mRNA decapping enzyme	84,397	85,068	+	672	85.7	EPTV-091
HYPV-84	mRNA decapping enzyme	85,043	85,822	+	780	92.0	EPTV-092
HYPV-85	ATPase, NPH1	85,796	87,703	−	1908	98.1	EPTV-093
HYPV-86	mRNA capping enzyme small subunit	87,746	88,609	−	864	96.5	EPTV-094
HYPV-87	Trimeric virion coat protein	88,643	90,295	−	1653	94.6	EPTV-095
HYPV-88	Late transcription factor (VLTF-2)	90,321	90,776	−	456	93.4	EPTV-096
HYPV-89	Late transcription factor (VLTF-3)	90,805	91,479	−	675	100.0	EPTV-097
HYPV-90	S-S bond formation pathway protein	91,476	91,706	−	231	93.4	EPTV-098
HYPV-91	P4b precursor	91,726	93,726	−	2001	93.3	EPTV-099
HYPV-92	RNA polymerase subunit (RPO19)	94,462	94,992	+	531	87.5	EPTV-101
HYPV-93	Virion morphogenesis core protein	94,989	96,107	−	1119	94.1	EPTV-102
**HYPV-94**	**Early transcription factor large subunit (VETF-L)**	**96,131**	**98,275**	**−**	**2145**	**97.8**	EPTV-103
HYPV-95	Intermediate transcription factor (VITF-3s)	98,338	99,213	+	876	94.2	EPTV-104
HYPV-96	IMV membrane protein, early morphogenesis	99,223	99,459	−	237	92.5	EPTV-105
**HYPV-97**	**P4a precursor**	**99,460**	**102,192**	**−**	**2733**	**90.5**	EPTV-106
HYPV-98	Viral membrane formation	102,207	103,142	+	936	96.1	EPTV-107
HYPV-99	Virion core and cleavage processing protein	103,139	103,705	−	567	76.7	EPTV-108
HYPV-100	IMV membrane protein, virion maturation	103,799	104,002	−	204	71.6	EPTV-109
HYPV-101	IMV membrane protein, essential	104,067	104,348	−	282	96.8	EPTV-110
HYPV-102	IMV membrane protein, non-essential	104,365	104,526	−	162	98.1	EPTV-111
HYPV-103	Core protein	104,516	104,809	−	294	95.9	EPTV-112
HYPV-104	Myristylated protein, essential for entry	104,793	105,935	−	1143	91.8	EPTV-113
HYPV-105	IMV membrane protein	105,936	106,526	−	591	96.9	EPTV-114
HYPV-106	DNA helicase, transcript release factor	106,541	107,995	+	1455	90.1	EPTV-115
HYPV-107	Zn finger-like protein, late morphogenesis	107,967	108,188	−	222	93.2	EPTV-116
HYPV-108	IMV membrane protein, entry|fusion	108,189	108,533	−	345	95.6	EPTV-117
HYPV-109	DNA polymerase processivity factor	108,532	109,809	+	1278	89.4	EPTV-118
HYPV-110	Holliday junction resolvase	109,793	110,338	+	546	91.3	EPTV-119
HYPV-111	Intermediate transcription factor (VITF-3L)	110,335	111,495	+	1161	91.5	EPTV-120
**HYPV-112**	**RNA polymerase subunit (RPO132)**	**111,492**	**115,010**	**+**	**3519**	**97.7**	EPTV-121
HYPV-113	A-type inclusion protein	114,996	117,869	−	2874	77.4	EPTV-122
HYPV-114	P4c precursor	117,926	119,800	−	1875	82.1	EPTV-123
HYPV-115	IMV membrane protein, fusion	119,856	120,206	−	351	86.2	EPTV-124
HYPV-116	IMV membrane protein, entry	120,207	120,623	−	417	94.2	EPTV-125
HYPV-117	RNA polymerase subunit (RPO35)	120,637	121,539	−	903	94.7	EPTV-126
HYPV-118	IMV protein	121,523	121,750	−	228	92.0	EPTV-127
HYPV-119	Hypothetical protein	121,953	122,435	+	483	80.7	EPTV-128
HYPV-120	ATPase|DNA packaging protein	122,465	123,235	−	771	95.3	EPTV-129
HYPV-121	C-type lectin-like EEV membrane phosphoglycoprotein	123,371	123,922	+	552	77.2	EPTV-130
HYPV-122	C-type lectin like IEV|EEV membrane glycoprotein	123,970	124,470	+	501	91.0	EPTV-131
HYPV-123	MHC class II antigen presentation inhibitor	124,509	125,033	+	525	81.0	EPTV-132
HYPV-124	Concanavalin-like precursor	125,073	125,918	+	846	78.0	EPTV-133
HYPV-125	EEV glycoprotein	125,953	126,681	+	729	68.7	EPTV-134
HYPV-126	Hypothetical protein	126,724	127,554	+	831	79.4	EPTV-135
HYPV-127	Hypothetical protein	127,578	127,817	+	240	68.2	EPTV-136
HYPV-128	Truncated CD47-like protein, integral membrane protein	127,814	128,404	−	591	80.3	EPTV-137
HYPV-129	Myristylated protein	128,422	128,829	+	408	73.3	EPTV-138
HYPV-130	Hypothetical protein	128,826	129,587	+	762	78.3	EPTV-139
HYPV-131	Chemokine binding protein	129,575	130,438	−	864	69.0	EPTV-140
HYPV-132	Profilin-like protein, ATI-localized	130,558	130,959	+	402	98.5	EPTV-141
HYPV-133	Hypothetical protein	130,956	131,339	−	384	76.9	EPTV-142
HYPV-134	3 beta-hydroxysteroid dehydrogenase|delta 5->4 isomerase	131,948	133,015	+	1068	84.2	EPTV-144
HYPV-135	Thymidylate kinase	133,646	134,233	+	588	85.2	EPTV-147
HYPV-136	DNA ligase-like protein	134,265	135,944	+	1680	87.2	EPTV-148
HYPV-137	A52R-like family protein	137,441	138,046	+	606	77.9	EPTV-150
HYPV-138	Hypothetical protein	138,641	139,717	+	1077	65.2	EPTV-151
HYPV-139	Toll|IL-1 receptor-like protein, IL-1, NFkB signaling inhibitor	139,781	140,428	+	648	89.8	EPTV-153
HYPV-140	Hypothetical protein	140,538	140,954	−	417	70.5	EPTV-154
HYPV-141	BTB kelch-domain protein	141,056	142,690	+	1635	76.8	EPTV-155
HYPV-142	Hemagglutinin	142,737	143,477	+	741	70.2	EPTV-156
HYPV-143	Ser|Thr protein kinase	143,532	144,467	+	936	91.0	EPTV-157
HYPV-144	IL-1 receptor antagonist	144,505	145,470	+	966	71.5	EPTV-158
HYPV-145	RING finger protein, host range	145,503	146,378	+	876	68.4	EPTV-159
HYPV-146	Partial schlafen-like protein	146,424	147,011	+	588	81.1	EPTV-160
HYPV-147	EEV type-1 membrane glycoprotein	147,108	147,815	+	708	75.3	EPTV-161
HYPV-148	Anti-apoptotic Bcl-2-like protein	147,851	148,294	+	444	73.5	EPTV-162
HYPV-149	Serpin 1	149,551	150,555	+	1005	76.9	EPTV-164
HYPV-150	Hypothetical protein	150,662	151,132	+	471	69.4	EPTV-165
HYPV-151	Tyrosine protein kinase-like protein	151,173	152,036	+	864	77.7	EPTV-166
HYPV-152	IL-1 beta-receptor	152,065	153,093	+	1029	71.9	EPTV-167
HYPV-153	Ankyrin repeat protein	153,125	155,104	+	1980	68.2	EPTV-168
HYPV-154	Ankyrin repeat protein	157,276	157,827	+	552	60.6	EPTV-169
HYPV-155	Alpha-amanitin target protein	158,306	159,013	+	708	82.3	EPTV-170
HYPV-156	NFkB inhibitor	159,072	159,752	+	681	81.1	EPTV-171
HYPV-157	Endothelin precursor	159,796	160,020	+	225	64.9	EPTV-172
HYPV-158	NFkB inhibitor	160,059	160,712	+	654	65.0	EPTV-173
HYPV-159	Secreted complement binding protein C3b|C4b	160,747	161,529	+	783	74.6	EPTV-174
HYPV-160	IL-18 binding protein	162,096	162,521	+	426	83.2	EPTV-175
HYPV-161	Ankyrin repeat protein	164,706	166,571	+	1866	60.3	EPTV-179

+/−: direction of open reading frame (ORF) (“+” (5′–3′) or “−” (3′–5′)).
